# Inhibitory activities of monoclonal antibodies against *Staphylococcus aureus* clumping factor A

**DOI:** 10.1128/mbio.02197-25

**Published:** 2025-09-08

**Authors:** Biswarup Banerjee, Carla Emolo, Miaomiao Shi, Abrar Abdullah Al Fardan, Tonu Pius, Muhammad Shafiul Azam, Molly McAdow, Olaf Schneewind, Dominique Missiakas

**Affiliations:** 1 Department of Microbiology, Howard Taylor Ricketts Laboratory, The University of Chicago, Lemont, Illinois, USA; University of California, Berkeley, Berkeley, California, USA

**Keywords:** monoclonal antibodies, *Staphylococcus aureus*, ClfA, blood stream infection

## Abstract

*Staphylococcus aureus* infection is a frequent cause of sepsis in humans, a disease associated with high mortality and without specific intervention. Clumping factor A (ClfA) displayed on the bacterial surface plays a key role in promoting *S. aureus* replication during invasive disease. Decades of research have pointed to a wide array of ligands engaged by ClfA. The sum of these interactions supports the unique ability of this pathogen to survive and replicate in the blood stream. One such ligand is fibrin. ClfA acts as the key agglutinating factor of *S. aureus* by promoting the shielding of bacteria in fibrin cables and their physical escape from phagocytes. Here, we compare a series of monoclonal antibodies elicited against the ligand binding domain of ClfA following immunization of mice. We analyze these antibodies for their ability to neutralize ClfA interactions and to promote the uptake of staphylococci in whole blood. We find that while all the antibodies promoted opsonophagocytic uptake, only those that also inhibited ClfA interactions with ligands reduced bacterial burdens in animals following blood stream challenge with *S. aureus*.

**IMPORTANCE:**

Antibody-based approaches to fight bacterial pathogens have been modeled on toxin-producing or encapsulated pathogens for which correlates of protection can be reduced to measuring antibody neutralization or complement-fixing activities using tissue-cultured cells. Such approaches have failed against *Staphylococcus aureus* raising uncertainty about the value of antibodies. Here, we use a series of mouse monoclonal antibodies directed against clumping factor A, a surface protein that allows *S. aureus* to thrive in the blood stream, to query how antibodies may be exploited against a pathogen endowed with a formidable array of virulence factors.

## INTRODUCTION

*Staphylococcus aureus* is a human pathogen that resides on the skin and nares but has the unique ability to replicate in the blood stream to cause mild to life-threatening infections (1, 2). Spillover to animals in close contact to humans and genetic adaptation also result in anthropogenic infections; as an example, *S. aureus* is a major cause of bovine mastitis in dairy cattle (3–5). *S. aureus* relies on surface proteins to breach host barriers and escape host defenses (6, 7). In *S. aureus*, most surface proteins are covalently linked to peptidoglycan by sortase enzymes (8, 9). Mutants lacking the housekeeping sortase A enzyme have been found to be avirulent in animal models (10, 11). Although sortase A anchors about 24 proteins on the bacterial surface, the exact count varying by strain (8, 9), clumping factor A (ClfA) has been shown to be critical for infection (12). Experiments have shown that animals are more likely to survive an intravenous *S. aureus* infection when challenged with *clfA* mutant strains (11, 13). ClfA mediates substrate interactions that enhance *S. aureus* adhesion to vascular epithelia and catheter-induced thrombi, facilitate *S. aureus* escape from neutrophils, and promote invasion into tissues including joints (14–18). Thus, ClfA is important in promoting *S. aureus* bacteremia, septic death, septic arthritis, endocarditis, and abscess formation in deep-seated organs (11, 13, 19–22). As a result, ClfA has emerged as a prime antigen target for the development of immune-based therapies in both humans and domestic animals, the latter in an attempt to reduce the incidence of bovine mastitis (19, 23–32).

**Fig 1:**
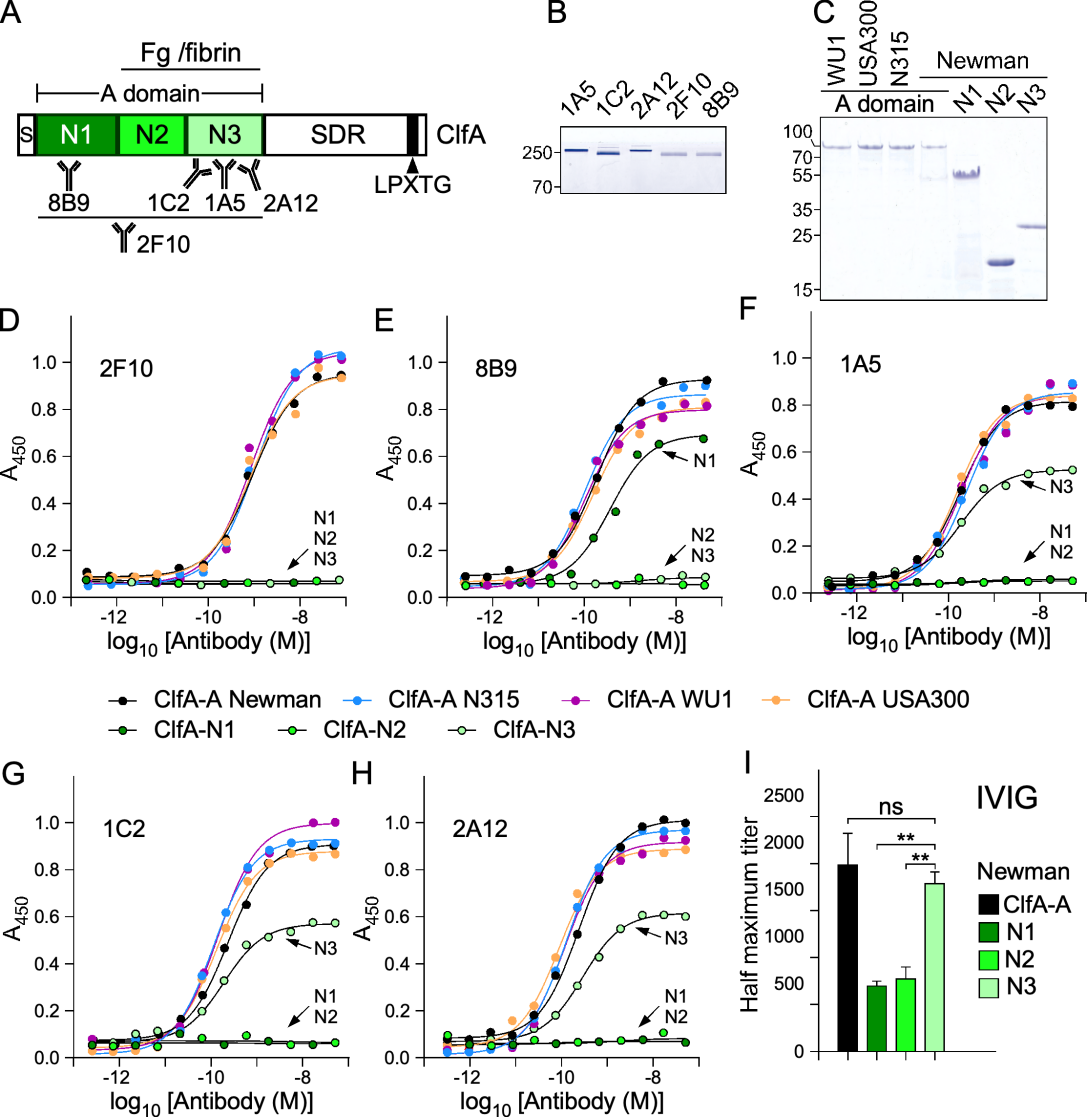
Characterization of anti-ClfA monoclonal antibodies (mAbs). (**A**) Domain organization of ClfA depicting the signal sequence (S), N1, N2, N3 subdomains, A domain (ClfA-A), serine aspartic repeat region (SDR), LPXTG motif for attachment to peptidoglycan by sortase A (black rectangle). N2-N3 segment involved in fibrinogen (Fg)/fibrin binding and binding sites of mAbs deduced from this study are shown. (**B, C**) Coomassie-stained gels of purified mAbs (non-reducing conditions) (**B**) and ClfA domains and subdomains from various strains (**C**). Numbers to the left of gels indicate molecular weight markers in kDa. (**D–H**) Representative enzyme-linked immunosorbent assay showing mAb binding to ClfA-A Newman, N315, WU1, USA300 as well as isolated, ClfA-N1, -N2 or -N3 subdomains from strain Newman. Antibodies are presented as follows: 2F10 (**D**), 8B9 (**E**), 1A5 (**F**), 1C2 (**G**), 2A12 (**H**). Bound antibodies were detected with polyclonal anti-mouse Horseradish peroxidase (HRP)-conjugated secondary antibody and reported as absorbance at 450 nm (*A*_450_). Association constants and comparative analyses are shown in Table 1 and Table S1. (**I**) Relative abundance of Newman ClfA-A, N1, N2, and N3 antibodies in intravenous immunoglobulin (IVIG). Data were analyzed with GraphPad Prism 10 using non-linear fit (least squared) regression. (**, *P* < 0.01; ns, not significant). All experiments were performed at least twice.

ClfA is a member of the Clf-Sdr-FnBP subfamily of microbial surface components recognizing adhesive matrix molecules (MSCRAMMs) (12). MSCRAMMs are characterized by the presence of two adjacent IgG-like subdomains responsible for substrate binding (12). *Staphylococcus epidermidis* SdrG interaction with the β-chain of fibrinogen exemplifies a binding mechanism described as “dock-lock-latch” and involves a trench between the two IgG-like subdomains (33, 34). In ClfA, the IgG-like subdomains are encompassed by the N2-N3 segment in the N-terminal A domain (ClfA-A) (35) (Fig. 1A); the hydrophobic trench between N2 and N3 captures the last 17 residues of the γ-chain of fibrinogen, and a conformational change at the C-terminus of N3 locks the peptide in place (36, 37). Fibrinogen is a glycoprotein (~340 kDa) composed of three pairs of covalently linked Aα-, Bβ-, and γ-chains with the letters A and B designating the fibrinopeptides released by thrombin cleavage (38). In the soluble dimer, the N-termini of all six chains form the central E domain, and each trimer extends at opposite ends through coiled-coil segments to form two symmetrical D domains composed mainly by the C-termini of the Bβ- and γ-chains (38). This symmetry accounts for the clumping activity of fibrinogen when added to *S. aureus* bacterial suspensions and the naming of ClfA (36, 39). But two decades of research have unveiled additional ClfA ligands. Recombinant ClfA has been shown to bind complement factor I, bovine annexin 2, and secreted *S. aureus* von Willebrand binding protein (vWbp), highlighting the pleiotropic roles of ClfA during infection (15, 17, 18, 40). Importantly, ClfA also promotes agglutination, the interaction of bacteria with fibrin, the insoluble form of fibrinogen produced during blood clotting, a process hijacked by the secreted coagulases of *S. aureus*, coagulase (Coa) and vWbp (13, 41).

Here, we isolated a series of monoclonal antibodies (mAbs) by immunizing mice with ClfA-A and characterized these antibodies for their ability to block *S. aureus* interactions with host ligands, promote opsonophagocytosis of bacteria in freshly drawn blood, and protect mice in a model of blood stream infection. Our findings suggest that the ability to block ClfA interactions with ligands, but not the simple opsonophagocytic activity of antibodies, is critical for protecting animals following a blood stream challenge. Thus, carefully designed antibodies targeting ClfA have the potential to reduce the severity of invasive disease associated with *S. aureus* bacteremia.

## RESULTS

### Characterization of mAbs raised against ClfA-A

For the purpose of this study, the A domain of *S. aureus* Newman ClfA, ClfA-A was used to immunize mice and isolate mAbs (42). The amino acid sequence of this antigen, residues 40–559, is shown in Fig. S1A. The antigen was produced in *Escherichia coli* without its cleavable signal sequence (residues 1–39) or repeats of serine aspartic (SD) amino acids (residues 560–668) that otherwise tether the A domain to the C-terminal sorting signal (residues 669–933) for attachment to the cell wall (Fig. 1A). In *S. aureus*, the SD repeats are extensively modified with N-acetyl-glucosamine and are not thought to bind any ligand (43, 44). Earlier efforts to develop a ClfA-based human vaccine (45, 46) and mAbs (28, 47) hinted to sequence variability among staphylococcal isolates. To evaluate this variability, we compared the sequences of some commonly studied isolates by our group, Newman, USA300 LAC (USA MRSA; [48]), N315 (Japanese MRSA; [49]), and WU1, a mouse-adapted strain used to study *S. aureus* colonization (50, 51) (Fig. S1A). We found that the ClfA-A sequences of strains Newman and USA300 LAC are identical with the exception of one substitution in the N1 subdomain (Fig. S1A). Greater variability exists between the ClfA-A sequences of strains N315 and WU1 (Fig. S1A). Next, a two-step local alignment strategy was performed against the non-redundant (nr) protein database to retrieve 3,367 ClfA-A sequences that were subjected to an all-against-all BLASTp comparison. A sequence similarity network derived from the resulting alignment revealed 58 distinct clusters (Fig. S1B). One representative sequence from each cluster was randomly selected and aligned using Clustal Omega, and the multiple sequence alignment visualized using ESPript 3.0 (Fig. S1C). This exercise revealed that the N3 domain is the most conserved of all three domains, and most importantly, residues involved in fibrinogen binding are absolutely conserved among all examined ClfA sequences (Fig. S1C). Thus, ClfA-A from strain Newman is a good representative for our experiment.

Following immunization of mice, five antibodies reactive to ClfA-A Newman were identified and purified (Fig. 1B) and characterized for their specificity and affinity to the full-length A domain as well as the separated N1, N2, and N3 subdomains of strain Newman (Fig. 1C). The various antigens were coated on 96-well plates and subjected to enzyme-linked immunosorbent assay (ELISA) using serial dilutions of each mAb (Fig. 1Dthrough H). mAb 2F10 was found to interact only with the full-length A domain but not with the isolated N1, N2, or N3 subdomains (Fig. 1D). The remaining antibodies bound the isolated N1 subdomain, mAb 8B9 (Fig. 1E), and the isolated N3 subdomain, mAbs 1A5, 1C2, 2A12, respectively (Fig. 1F through H; Table 1). When binding was detected, the half-maximal effective concentration (EC_50_) values were not found to be statistically significant in any pairwise comparison (Table S1), meaning that all mAbs bound the ligands with similar affinity.

**TABLE 1:** Interactions between mAbs and various ClfA antigens as determined by ELISA^*a*^

Antibody/antigen	EC_50_ ± SEM (×10^−9^ M)						
	N1	N2	N3	ClfA-A Newman	ClfA-A N315	ClfA-A WU1	ClfA-A USA300
1C2-mIgG1	/	/	1.8 ± 1.8	2.2 ± 1.1	1.1 ± 1.6	1.3 ± 1.5	1.8 ± 1.3
2F10-mIgG1	/	/	/	1.3 ± 0.3	1.2 ± 0.03	1.5 ± 0.02	1.5 ± 0.01
8B9-mIgG1	2.6 ± 1.8	/	/	2 ± 1.5	1.2 ± 1.5	1.1 ± 1.5	1.8 ± 1.6
mIgG1	/	/	/	/	/	/	/
1A5-mIgG2a	/	/	1.9 ± 1.5	1.9 ± 1.6	2.6 ± 0.73	1.6 ± 1.6	1.9 ± 1.6
mIgG2a	/	/	/	/	/	/	/
2A12-mIgG2b	/	/	2.7 ± 0.6	2.2 ± 1.2	1.7 ± 1.6	1.1 ± 1.7	1 ± 1.8
mIgG2b	/	/	/	/	/	/	/

^*a*^ Antigens were immobilized on plates for ELISA measurements. Three independent experimental determinations were performed. Data were used to derive the half-maximal effective concentrations (EC_50_) with SEM for each test mAb. The symbol “/” indicates that no binding was detected. All other values were analyzed with one-way ANOVA with Dunnett's multiple comparison test between each test article and each antigen. No significant differences were found (analysis shown in Table S1).

To assess cross-reactivity, the ClfA-A domains of strains USA300 LAC, N315, and WU1 were cloned as described for strain Newman and purified to homogeneity (Fig. 1C). ELISA experiments revealed that each of the five mAbs interacted similarly with these ClfA-A variants regardless of their allelic differences (Fig. 1D through H; Table 1; Table S1). ELISA was also used to determine the isotype of the five mAbs and revealed that 1C2, 2F10, and 8B9 belong to the mIgG1 subclass, 1A5 belongs to the mIgG2a subclass, and 2A12 to the mIgG2b subclass (Fig. S2). Since three mAbs interacted with N3, a competition ELISA was performed with Horseradish peroxidase (HRP) labeled 2A12 and unlabeled 1A5, 1C2, and 2A12. This approach revealed that 1A5 and 2A12 may bind the same epitope (Fig. S3).

Three of our mAbs recognized the N3 subdomain, a property also shared with tefibazumab, a humanized antibody derived from the mouse hybridoma MAb 12-9 (47). Our hybridomas were generated using the same antigen, mouse line, and immunization protocol (47). We wondered if this approach may have introduced some bias toward N3 recognition. To examine this possibility, commercially available intravenous immunoglobulin (IVIG) was assessed for the presence of antibodies against ClfA-A, as well as the isolated N1, N2, and N3 subdomains. This analysis revealed that the N3 immunodominance is not restricted to mice as ClfA antibodies in human preferentially bind N3 (Fig. 1I).

### Assessing mAbs for the disruption of ClfA interaction with human fibrinogen and fibrin

**Fig 2:**
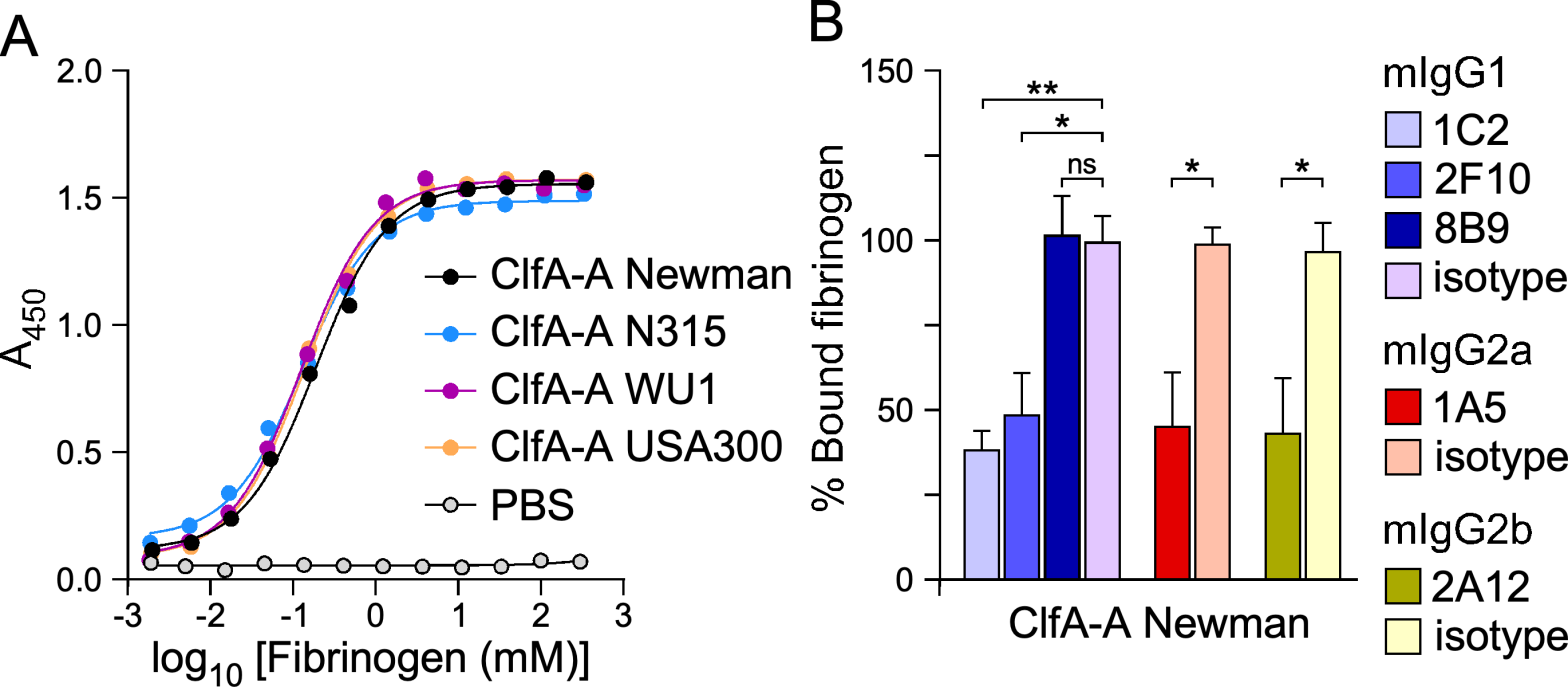
mAb inhibition of ClfA-A interaction with soluble fibrinogen. (**A**) Association of soluble fibrinogen suspended in phosphate buffered saline (PBS) with immobilized ClfA-A proteins from various strains. Bound fibrinogen was detected with polyclonal anti-human fibrinogen-HRP and reported as *A*_450_. PBS was used as a negative control. Half-maximal effective concentrations (EC_50_) are reported in Table 2. (**B**) The ability of ClfA antibodies to compete for ClfA-A/fibrinogen interactions was measured using ELISA. Half-maximal inhibitory concentrations (IC_50_) are reported in Table 3. This panel shows the percentage of fibrinogen remaining bound to ClfA-A in the presence of 25 nM of antibodies. *A*_450_ values of bound fibrinogen in the presence of isotype control antibodies were set at 100%. Data are represented as mean ± SEM, and statistical significance was calculated using one-way ANOVA with Tukey’s multiple comparison (**, *P* < 0.01; *, *P* < 0.05; ns, not significant). All experiments were performed at least twice.

ClfA mediates *S. aureus* interaction with fibrinogen, and all ClfA-A variants used in this study interacted with similar affinity with human fibrinogen (Fig. 2A; Table 2). This was somewhat unsurprising, given the strict conservation of ClfA residues interacting with the C-terminus of the γ-chain of fibrinogen as shown in Fig. S1C. Next, fibrinogen was mixed with serial dilutions of mAbs before adding to immobilized ClfA-A. This approach showed that all the mAbs, with the exception of 8B9 (with N1 specificity), inhibited ClfA-A interaction with fibrinogen in a concentration-dependent manner and regardless of differences in amino acid sequences (Table 3); this competition was also depicted as the fraction of fibrinogen that remains bound to ClfA-A Newman in the presence of 25 nM of mAbs (Fig. 2B).

**TABLE 2:** Interactions between various host and bacterial ligands

Human ligand	EC_50_ ± SEM (×10^−6^ M)^*a*^						
	ClfA-A Newman	ClfA-A N315	ClfA-A WU1	ClfA-A USA300	vWbp Newman	vWbp + ClfA-A Newman	
Fibrinogen	1.3 ± 0.01	1.3 ± 0.08	1.1 ± 0.02	1 ± 0.09	ND	ND	
von Willebrand factor	/	ND	ND	ND	14.5 ± 1.69^*b*^	3.5 ± 0.35^*b*^	
Complement factor I	1.8 ± 0.3	ND	ND	ND	ND	ND	

^*a*^ Data were used to derive the half-maximal effective concentrations (EC_50_) with SEM. Three independent experimental determinations were performed using ELISA. The symbol “/” indicates that no binding was detected. ND indicates that the experiment was not determined. When analyzed with one-way ANOVA with Dunnett's multiple comparison test, no significant differences were found between ClfA-A molecules and fibrinogen binding; however, inclusion of ClfA-A significantly increased the interaction between von Willebrand factor and vWbp. All statistical analyses are shown in Table S1.

^*b*^ These two values were compared with the unpaired *t* test, *P *= 0.0237.

**TABLE 3:** Inhibition of ClfA-A interaction with various ligands by mAbs^*a*^

mAb	IC_50_ ± SEM (×10^−10^ M)					
	Fibrinogen				von Willebrand factor	Complement factor I
	ClfA-A Newman	ClfA-A N315	ClfA-A WU1	ClfA-A USA300	ClfA-A + vWbp Newman	ClfA-A Newman
1C2-mIgG1	1.8 ± 0.4	1.3 ± 0.7	1.6 ± 0.4	2 ± 1.3	2.5 ± 0.3	/
2F10-mIgG1	1.2 ± 0.7	1.1 ± 0.3	1 ± 0.6	1.1 ± 0.4	5.7 ± 0.2	/
8B9-mIgG1	/	/	/	/	/	/
mIgG1	/	/	/	/	/	/
1A5-mIgG2a	1 ± 0.58	1 ± 1	1 ± 0.7	1.2 ± 1.1	8.1 ± 0.43	/
mIgG2a	/	/	/	/	/	/
2A12-mIgG2b	1.3 ± 0.3	1.9 ± 0.2	1.3 ± 1	1.1 ± 0.7	3.6 ± 0.2	/
mIgG2b	/	/	/	/	/	/

^*a*^ Three independent experimental determinations were performed using ELISA to derive half-maximal inhibitory concentrations (IC_50_) with SEM for each mAb. The symbol “/” indicates that the ClfA-A interaction with ligand was not inhibited upon addition of antibody. All statistical analyses are shown in Table S2.

Structural studies have shown that the Fab region of tefibazumab binds the head portion of N3 (residues involved in this interaction are shown in Fig. S1C), not the N2-N3 interface that captures the γ-chain of fibrinogen (36, 37), suggesting that ClfA may have two fibrinogen-binding sites (52). We asked if pairwise combinations of non-overlapping 2A12 and 1C2 or combinations with our in-house-produced tefibazumab (53) may block fibrinogen binding better than single mAbs. However, this was not the case (Fig. S4A), perhaps steric hindrance by a single antibody is sufficient to block both binding sites. Next, we used isolated ClfA-A, N2, and N3 domains to purify ClfA antibodies from IVIG and found that only anti-ClfA-N3 IVIG (not anti-ClfA-N2 IVIG) antibodies inhibited ClfA-A interactions with fibrinogen in a manner that was similar to inhibition by mIgG2b-2A12 and anti-ClfA-A IVIG antibodies (Fig. S4B).

**Fig 3:**
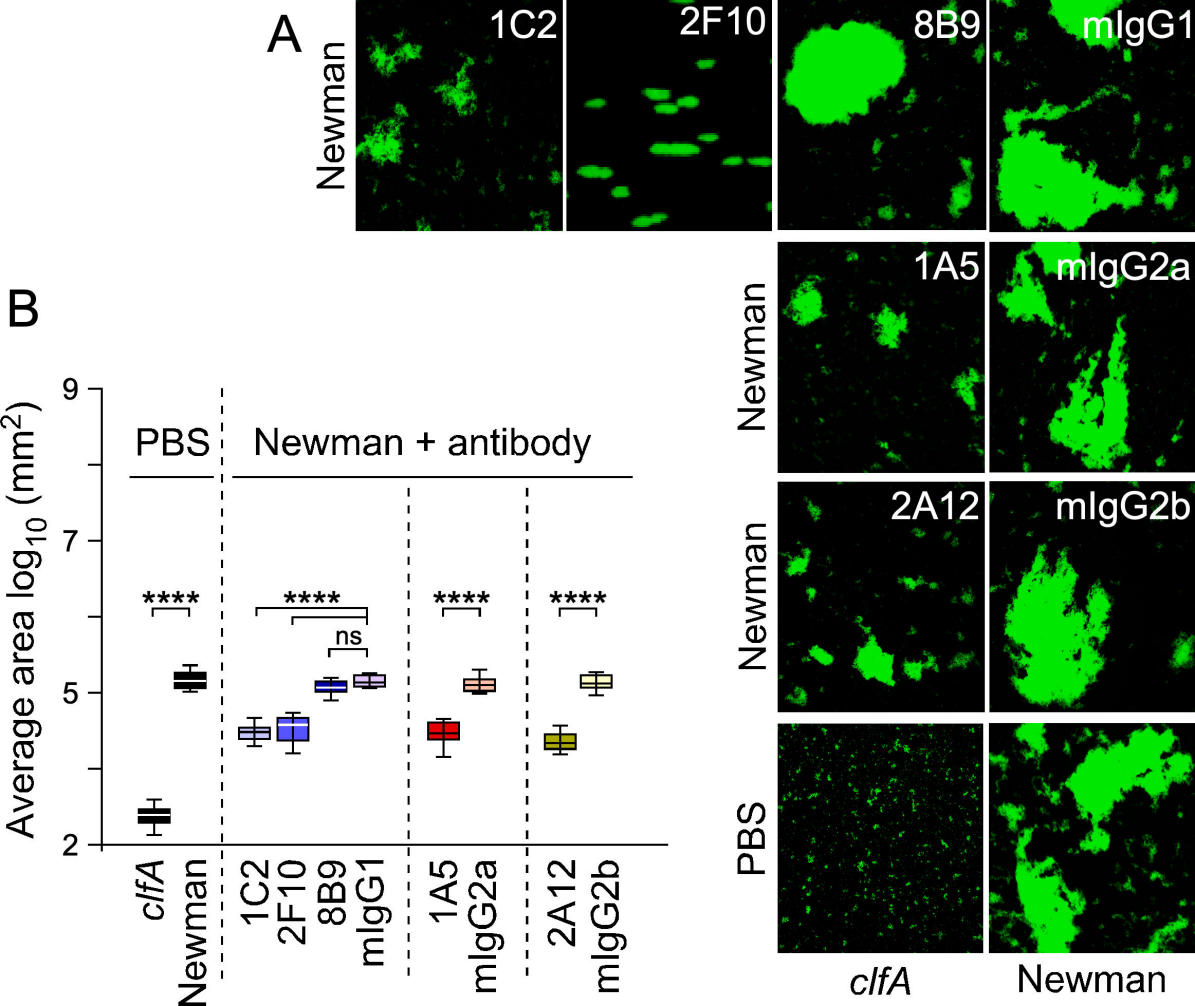
Staphylococcal agglutination in anti-coagulated mouse plasma. (**A**) Representative images of SYTO-9-stained Newman bacteria agglutinated in mouse plasma in the presence of isotype control or test antibodies that were obtained using an inverted fluorescent microscope with a 20× objective. (**B**) Box and whisker plot representation of agglutination areas with anti-coagulated mouse plasma collected from 10 fields of microscopic view. Statistical significance was assessed in pairwise comparison (wild-type Newman vs *clfA* mutant; isotype control vs test antibody) using Brown-Forsythe and Welch ANOVA followed by Dunnett’s T3 correction for multiple comparisons (****, *P* < 0.0001; ns, not significant). All experiments were performed at least twice.

Next, we asked if mAbs isolated in this study could inhibit ClfA-mediated agglutination of bacteria in anti-coagulated plasma (54). While clumping is the result of bacterial interaction with soluble fibrinogen (36, 39), *S. aureus* agglutination can be visualized as the assembly of large aggregates in a network of fibrin, a reaction that requires the host factors prothrombin and fibrinogen (54), as well as ClfA and secreted Coa and vWbp, the factors that bind and activate prothrombin (13, 55). Thus, agglutination represents a more physiological host-pathogen interaction encountered during bacteremia. *S. aureus* Newman bacteria were fluorescently stained with SYTO-9 and incubated with EDTA-chelated mouse plasma with mAbs or isotype control antibodies on a glass microscope slide for 30 min. Representative images shown in Fig. 3A were used to quantify the size of agglutinates under the various treatments (Fig. 3B). This approach revealed that all N3 (1A5, 1C2, and 2A12) and A domain-only binding (2F10) mAbs significantly reduced bacterial agglutination in plasma compared to cognate isotypes, but no reduction was observed for N1 binding, mAb 8B9 (Fig. 3). Isotype controls had no significant reduction in areas of agglutination compared to the positive control, Newman with no additional treatment (Fig. 3). The isogenic *clfA* mutant served as a negative control (Fig. 3). Together, these experiments demonstrate that mAbs that prevent ClfA-fibrinogen interaction also block *S. aureus* agglutination in plasma.

### mAbs reduce *S. aureus* adhesion by impeding ClfA-vWbp-vWF tripartite interaction

**Fig 4:**
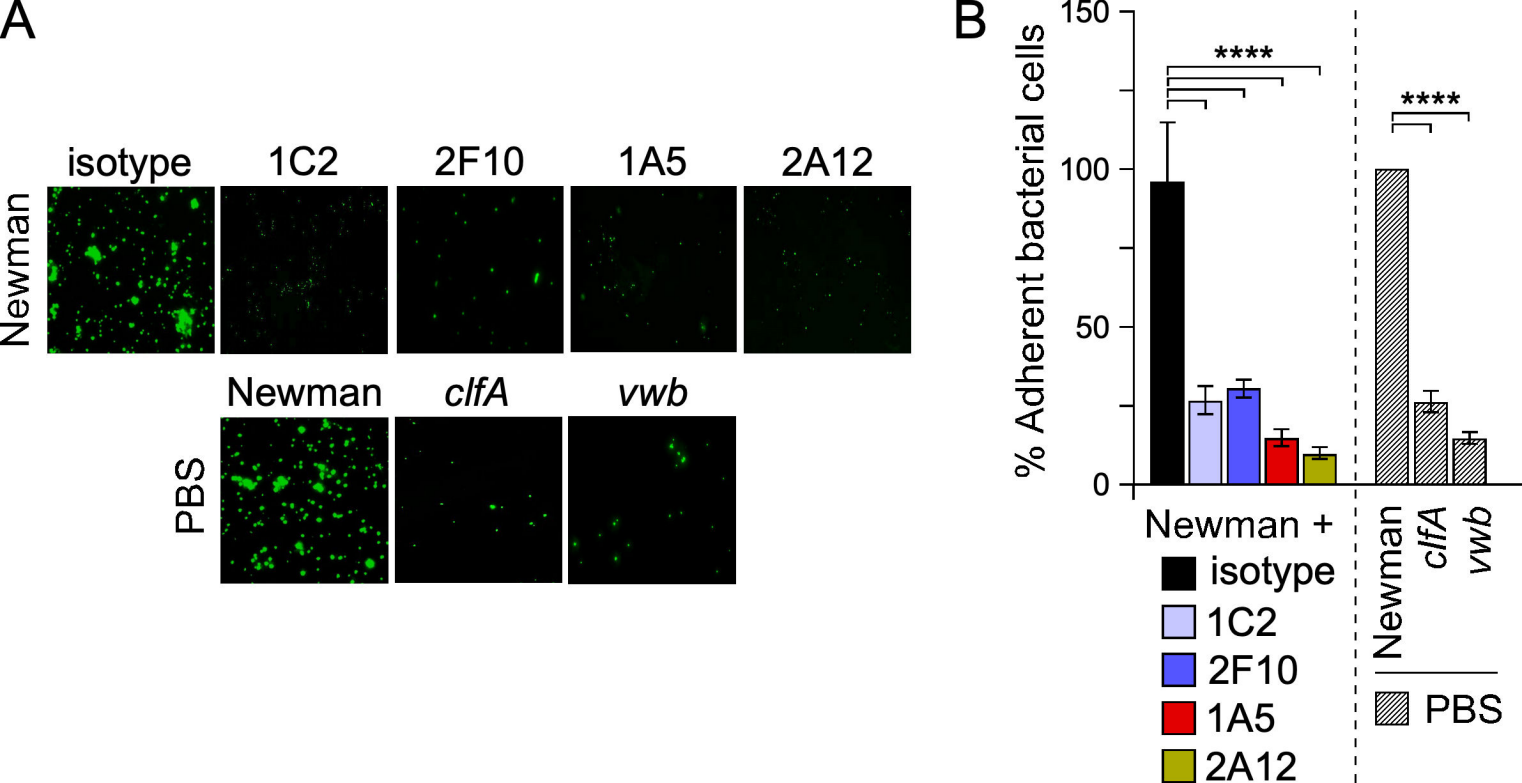
Staphylococcal adhesion to HUVECs under flow. (**A**) Representative images of SYTO-9-stained Newman bacteria that adhered to HUVEC cells under flow in iBidi microslide. Images were captured using an inverted fluorescent microscope with a 40× objective. (**B**) Quantification of adhesion as shown in panel A was performed by scanning fluorescence signals from 10 fields of microscopic view. Adhesion is plotted as percentage with 100% representing signals obtained for wild-type bacteria with no additional treatment (Newman/PBS). Data are displayed as mean ± SEM, and statistical significance was calculated using one-way ANOVA with Tukey’s multiple comparison (****, *P* < 0.0001). All experiments were performed twice for reproducibility.

In the vasculature, ClfA promotes *S. aureus* adhesion via a fibrinogen bridge between the bacterial cell and the α_V_β_3_ integrin on the endothelial cell surface (56). Thus, antibodies blocking fibrinogen interactions would also prevent such adhesion. Genetic and biochemical studies have also demonstrated that secreted vWbp interacts with ClfA to promote *S. aureus* adhesion under shear blood flow in a fibrinogen-independent manner (17, 18, 57). Shear stress unfolds secreted von Willebrand factor (vWF), unmasking sites for binding to the α_V_β_3_ integrin on endothelial cells of blood vessel walls (58). This unfolding also unmasks sites for both platelet and *S. aureus* adhesion (17). Like platelets, *S. aureus* binds directly to the A1 domain of vWF, utilizing an ultra-strong bond between ClfA and vWbp as measured using atomic force microscopy (17, 59). The weak interaction between purified vWbp and vWF was recapitulated in our ELISA and found to be significantly increased upon addition of ClfA-A (Table 2) confirming the earlier report (60). Addition of mAbs, with the exception of N1 binding mAb 8B9, disrupted the ClfA-A-mediated interaction between vWbp and vWF, in a dose-dependent manner (Table 3). To assess the impact of mAbs on *S. aureus* adhesion under flow more directly, fluorescently labeled bacteria were perfused at 1 mL/min over human umbilical vein endothelial cells (HUVECs) seeded in a chambered coverslip. HUVECs were activated with a Ca^2+^ ionophore to cause the release of vWF and bacterial attachment captured with a fluorescence microscope in the presence of anti-ClfA mAbs or isotype control. Representative images shown in Fig. 4A and quantified for number of bound bacteria (Fig. 4B) revealed that mAbs with inhibitory binding activity toward vWbp-vWF proteins (Table 3) also reduced *S. aureus* adhesion. Newman and isogenic *clfA* and *vwb* mutants with no additional treatments served as positive and negative controls (Fig. 4). Together, the results indicate that antibodies that inhibit fibrin and fibrinogen interactions also have the potential to block ClfA-mediated interactions critical for bacterial adhesion in the vasculature.

### mAbs promote opsonophagocytic killing of *S. aureus* in mouse blood

**Fig 5:**
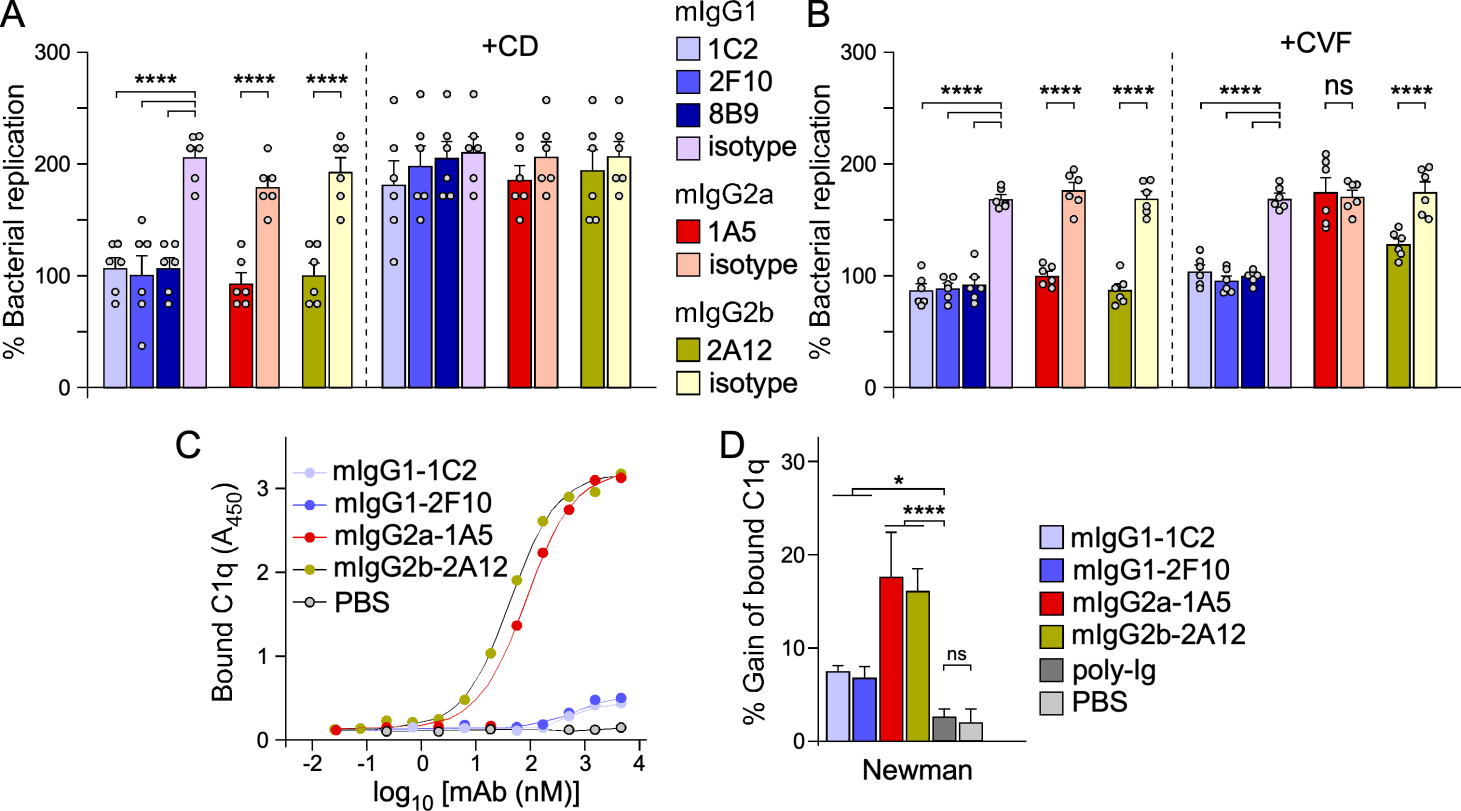
Opsonophagocytic and C1q binding activities of anti-ClfA antibodies. (**A, B**) Staphylococcal survival in freshly drawn mouse blood was assessed by inoculating mid-log phase bacteria (2.5 × 10^5^ CFU) into 0.5 mL of freshly drawn anti-coagulated mouse blood. At 0 and 30 min, buffer was added to lyse host cells and release bacteria trapped in fibrin or NETs. Bacteria were counted by plating serial dilutions on solid medium and reported as percentage of viable counts, with 100% representing bacterial counts at time 0. In panel A, one set of experiments was performed with blood pre-incubated with cytochalasin D to block phagocytic uptake. In panel B, one set of experiments was performed with blood pre-incubated with cobra venom factor to deplete complement. Data in each panel are from two independent experiments and are presented as mean ± SEM. Statistical significance was performed by Brown-Forsythe and Welch ANOVA followed by Dunnett’s T3 correction for multiple comparisons (****, *P* < 0.0001). Experiments were performed twice for reproducibility. (**C, D**) C1q binding. Binding of mouse C1q was examined with immobilized test antibodies or PBS (**C**) or with immobilized Newman bacteria pre-incubated with test antibodies, poly-Ig, or PBS (**D**). Bound C1q was detected using anti-mouse C1q-HRP, and signals recorded as *A*_450_. The *y*-axis in panel D represents gain in C1q binding above the PBS control; this quantification was achieved by taking each *A*_450_ value (including PBS with background C1q binding) and dividing by the average absorbance value of the PBS control multiplied by 100 (*A*_450_ values were obtained in triplicates for each treatment); the data are presented as mean ± SEM, and statistical significance calculated using one-way ANOVA with Tukey’s multiple comparison (****, *P* < 0.0001; *, *P* < 0.05; ns, not significant). Experiments in panels C and D were performed twice independently.

In addition to inhibiting ClfA-ligand interactions, we would expect that antibodies targeting a surface antigen would promote bacterial uptake and killing by phagocytic cells. We used freshly drawn anti-coagulated mouse blood supplemented with candidate or isotype control antibodies to evaluate the opsonophagocytic killing potential of mAbs. Since the agglutinating activity of ClfA shields staphylococci in fibrin cables, this approach is deemed more relevant than using purified neutrophils or differentiated HL-60 cells in an uptake assay (61). Bacteria were incubated in freshly drawn blood with antibodies for 30 min. Longer incubations lead to the deterioration of samples with extensive lysis of host cells. At the end of this incubation and before plating for colony forming enumeration (CFU), samples were treated with streptokinase and nucleases to liberate bacteria trapped in fibrin clots or neutrophil extracellular traps (NETs), and with saponin to release intracellular staphylococci not killed by immune cells. Counts at 30 min were reported as percentages, with 100% representing CFU counts at time 0. In one set of experiments, the assay was performed using blood pre-treated for 10 min with cytochalasin D (CD), an inhibitor of actin polymerization that prevents phagocytosis by host cells (Fig. 5A). This was done to document if antibodies promoted phagocytic uptake of bacteria. To query the requirement for complement, in a second set of experiments, blood was preincubated with cobra venom factor (CVF), a protein analog of complement component C3 that continuously activates complement resulting in its depletion (62) (Fig. 5B). We observed an approximately 50% reduction in bacterial counts in samples treated with the anti-ClfA mAbs compared to the respective isotype controls (Fig. 5A and B; left panels). No statistical differences were observed between candidate mAbs. Addition of CD eliminated any protective activity mediated by mAbs (Fig. 5A). Addition of CVF had no impact on the activity of 1C2/2F10/8B9-mIgG1 and 2A12-mIgG2b but preempted any protection mediated by 1A5-mIgG2a (Fig. 5B). These results agree with the notion that mIgG1 and mIgG2a display negligible and high interactions with C1q, respectively (Fig. 5C) (63, 64), a behavior that is corroborated when assessing mAb-mediated C1q recruitment on the surface of *S. aureus* (Fig. 5D). Thus, the loss of killing activity by 1A5-mIgG2a upon depletion of complement conforms with the intrinsic ability of this antibody to interact with C1q. But the finding that 2A12-mIgG2b was not abrogated in the presence of CVF is somewhat surprising (Fig. 5B) as this antibody did promote C1q recruitment on the surface of *S. aureus* (Fig. 5D). Another interesting finding is that mAb 8B9, which lacked any inhibitory activity toward ClfA-A ligands, is able to significantly promote the uptake of bacteria in whole blood, even in the absence of complement (Fig. 5A and B).

It has previously been shown that ClfA may also recruit complement factor I on the bacterial surface, thereby increasing the cleavage of C3b into inactive C3b, an activity shown to correlate with reduced phagocytosis of staphylococci by purified human neutrophils (15, 65, 66). Complement factor I is also known as the C3b/C4b inactivator. Using ELISA, we confirmed that ClfA-A interacts with complement factor I (Table 2); however, none of the mAbs was able to displace this interaction (Table 3).

### Anti-ClfA mAbs protect mice against blood stream challenges with *S. aureus*

**Fig 6:**
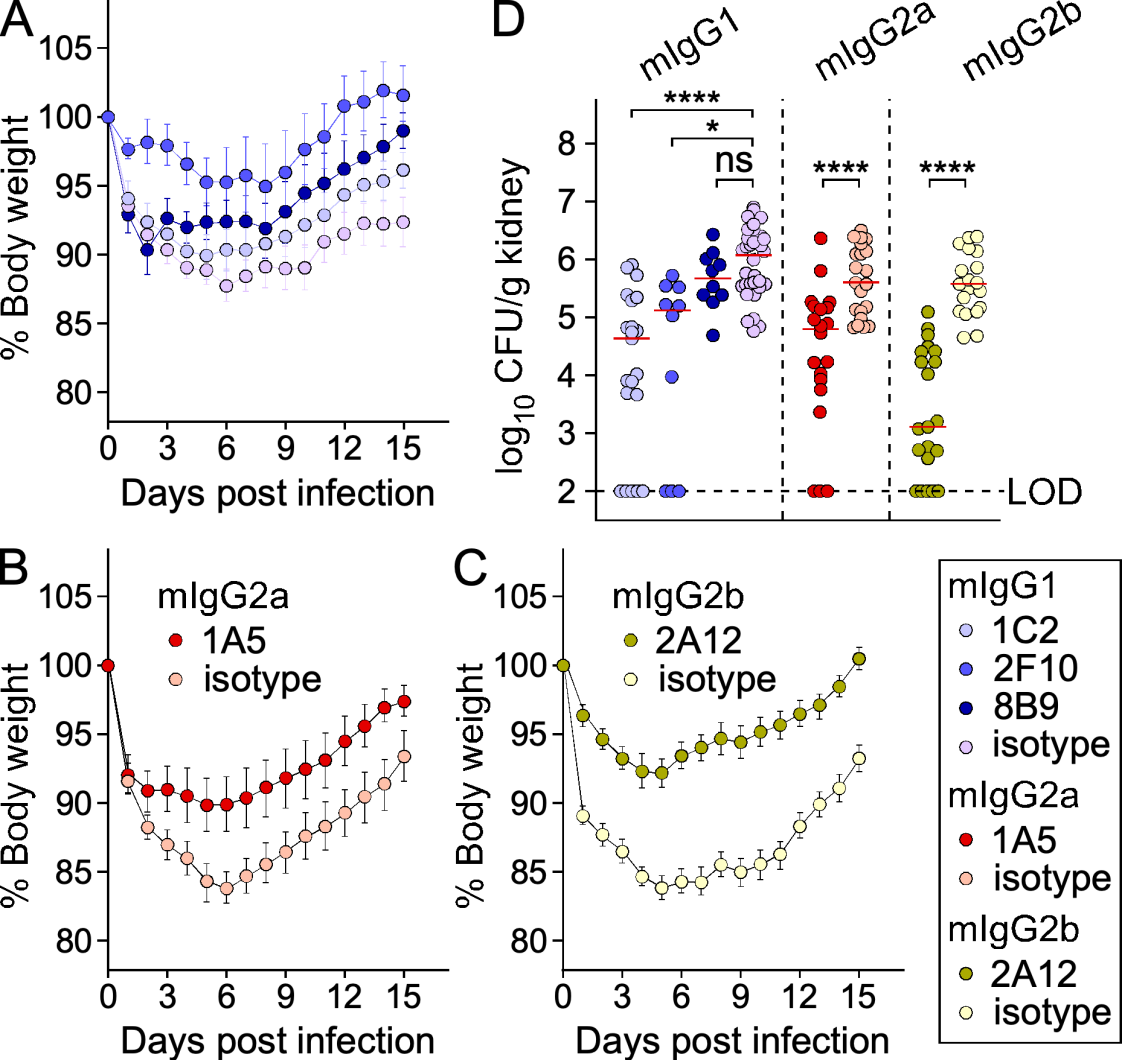
Protective activity of anti-ClfA mAbs following intravenous challenge of mice with *S. aureus.* Animals (*n* = 8–12) were passively immunized with test or control antibodies (5 mg/kg IP) and challenged 24 hours later with a sublethal inoculum of strain USA300. Mice were weighed daily. (**A–C**) Weights were recorded daily, averaged, and changes reported as percentage with 100% set on day 0 of the experiment (infection day). Data were split by isotypes for clarity. Statistical differences were calculated with the Kruskal-Wallis test followed by Dunn’s multiple test correction. (**D**) Bacterial loads in kidneys were enumerated by plating organs on day 15 post-infection. Bacterial counts are presented as median log_10_ CFU per gram of kidney (LOD, limit of detection). Statistical significance was calculated using one-way ANOVA (mixed-model) with Gaussier-Greenhouse correction followed by Tukey’s multiple comparison test (****, *P* < 0.0001; *, *P* < 0.05; ns, not significant). A cumulative of at least two independent challenge studies is shown (testing of the 8B9 antibodies was only performed twice).

A passive immunization experiment was performed to evaluate the protective activity of candidate mAbs in a mouse model of blood stream infection. Antibodies were administered 24 hours prior to intravenous challenge with a sublethal inoculum of *S. aureus*. In this model, bacteria that survive replication in blood disseminate to organ tissues to seed abscess lesions (11, 67, 68), a metastatic activity also observed following *S. aureus* bacteremia in humans (1, 2, 69). In infected mice, disease severity can be assessed by recording weight changes daily. As expected, significant weight losses were observed during the acute phase of disease (approximately first 6 days post-challenge) followed by a slow recovery (Fig. 6A through C). Animals were grouped by isotypes to better illustrate these changes. Of note, weight loss was less exacerbated with the passive administration of isotype control mIgG1 (Fig. 6A) as compared to mIgG2a (Fig. 6B) and mIgG2b (Fig. 6C) (Table S3). As a result, alleviation of weight loss was more significant upon administration of 1A5-mIgG2a (Fig. 6B) or 2A12-mIgG2b (Fig. 6C) (Table S3). Animals were killed on day 15 post-infection, and bacterial burdens in kidneys (an organ where bacteria continue to replicate in deep-seated abscesses for weeks) were enumerated by plating serial dilutions of homogenized tissues. Animals pre-treated with 1C2-mIgG1, 2F10-mIgG1, 1A5-mIgG2a, and 2A12-mIgG2b showed significantly lower bacterial load in kidneys compared to isotype controls (Fig. 6D). Although, treatment with 2A12-mIgG2b resulted in the greatest reduction in the median bacterial loads, this reduction was not statistically significant as compared to treatment with the other three mAbs (Table S3). Animals pre-treated with 8B9-mIgG showed no reduction in bacterial load in the kidneys of infected animals (Fig. 6D). Thus, although 8B9 displays opsonophagocytic activity in whole blood, this activity alone is not sufficient to reduce bacterial burdens in infected animals.

## DISCUSSION

*S. aureus* mutants lacking functional *clfA* display virulence defects in several mouse models of infection, including septic arthritis, endocarditis, and abscess formation, following blood stream dissemination of bacteria (11, 13, 19–22). These phenotypes have largely been correlated with the loss of staphylococcal binding to fibrinogen. Indeed, mice lacking the last five residues of the fibrinogen γ-chain (ClfA binding site) are more resistant to *S. aureus* sepsis (16, 70). Over 20 years ago, Hall and co-workers isolated mouse hybridomas MAb 12-9, MAb 35-052, and MAb 15EC6 using ClfA-A Newman as an immunogen (47). Only MAb 12-9, that bound ClfA on the surface of *S. aureus* and inhibited interaction with fibrinogen, conferred a modest survival benefit in BALB/c mice challenged intravenously with a lethal inoculum of strain Newman (47). Lack of protection by the other two antibodies was attributed to an inability to recognize ClfA on the surface of *S. aureus* (MAb 35-052) and to inhibit ClfA interaction with fibrinogen (MAb 15EC6), respectively (47). Here, we used the same antigen to isolate murine mAbs and exploited the N1, N2, and N3 subdomains to further delineate the specificity of mAbs. Of five antibodies identified in this study, three recognized the N3 subdomain, one the N1 subdomain, and one the full-length ClfA-A domain but not the isolated subdomains. We found that all the antibodies, with the exception of 8B9 (N1 specificity), were able to inhibit the interaction of ClfA with fibrinogen *in vitro*. However, the past 20 years of research have also revealed that ClfA binds to multiple ligands (12). In fact, it is now recognized that most MSCRAMMs have multiple ligands, allowing the limited repertoire of surface proteins to carry out a vast array of host-pathogen interactions (8, 9, 12). ClfA mediates interactions with fibrin, complement factor I, and vWbp and vWF. In turn, these interactions promote bacterial agglutination, escape from phagocytes and complement, and bacterial adhesion. We found that with the exception of 8B9 (N1 specificity), all the mAbs reduced bacterial agglutination and adhesion to host cells under flow, the latter inhibitory activity also correlating with the disruption of the ClfA-vWbp-vWF tether *in vitro*. However, none of the mAbs could prevent the interaction between complement factor I and ClfA-A. Whether neutralizing such an interaction is important for protection during blood stream infection remains to be determined.

Hall and co-workers restricted their selection of mAbs to mIgG1, reasoning that this isotype is not captured by staphylococcal protein A (SpA) on the surface of *S. aureus* (47). Indeed, SpA binds the Fc (fragment crystallizable) region of IgG with the hierarchy mIgG2a > mIgG2b > mIgG3 >>> mIgG1. SpA-mediated Fc binding diverts antibodies from their target and interferes with C1q recruitment, effectively preventing complement fixing and opsonophagocytosis of bacteria (71–73). mAbs examined in our study were of various subclasses, allowing us to correlate bacterial killing in blood and in animals with the purported effector functions of antibodies. Mouse IgG Fc regions interact with various affinities with C1q and Fcγ receptors (FcγRs) that include activating, FcγRI, FcγRIII, FcγRIV, and inhibitory, FcγRIIb, receptors (74, 75). For example, while mIgG1 does not bind C1q, it can interact with the activating FcγRIII and inhibitory FcγRIIb receptors (74–76). Conversely, mIgG2a and mIgG2b trigger complement fixing on target cells and promote interactions with all activating FcγRs (74–76). When calculated as the A/I ratio representing the highest binding affinity for an activating (A) over the inhibitory (I) receptor, mIgG2a has the highest A/I ratio, followed by mIgG2b and mIgG1 (A/I values of 69, 7, and 0.1, respectively) (75). When interrogated for their ability to promote bacterial killing in whole blood, all the mAbs in our study were found to enhance bacterial uptake, including 8B9-mIgG1. Addition of CVF in this assay did not alter the activity of mIgG1 antibodies 1C2, 2F10, and 8B9 but abrogated the activity of 1A5-mIgG2a. This agrees with the respective affinities of mIgG1 (negligible) and mIgG2a (high) toward C1q. Since the opsonophagocytic activity of 2A12-mIgG2b was not affected by CVF, we surmise that in this assay, 2A12 may operate in an FcγR-dependent manner. All five antibodies were also tested in a passive immunization study, whereby BALB/c mice were challenged intravenously with *S. aureus* and killed 15 days post-infection to enumerate bacteria in kidneys. This experiment revealed that treatment with all the antibodies, with the exception of 8B9-mIgG1, resulted in reduced bacterial burdens. Thus, it appears that the most effective mAbs have two activities: they neutralize ClfA interactions with host ligands and promote opsonophagocytic uptake of bacteria. In case of 2F10 and 1C2, it is also possible that some protection may have been contributed by the purported anti-inflammatory activity of the mIgG1 subclass (76, 77).

The mouse hybridoma MAb 12-9 was subsequently humanized using a human IgG1 backbone. These efforts yielded tefibazumab (78). A phase II clinical trial with bacteremic patients compared the efficacy of tefibazumab (Aurexis) and antibiotic treatment with placebo (24). However, composite clinical end point analysis did not detect differences between placebo and antibody (24). Two reasons were put forward to account for this clinical failure. It was purported that tefibazumab binding to the head portion of N3 instead of the N2-N3 interface of ClfA might have been suboptimal in inhibiting fibrinogen binding; furthermore, tefibazumab may have failed to recognize ClfA variants from diverse clinical isolates (52). However, an extensive sequence alignment of ClfA proteins suggests that tefibazumab interacts with highly conserved residues of ClfA (Fig. S1C). Furthermore, combining mAbs did not result in increased neutralizing (inhibitory) activity, and only anti-N3 antibodies purified from IVIG were found to inhibit ClfA-fibrinogen interactions. Anti-N3 antibodies were also the most abundant antibodies in IVIG, and N3 is the most conserved domain among ClfA isolates (Fig. S1C). Thus, lack of neutralizing activity and sequence variability of the antigen may not solely account for clinical failure. We favor the possibility that the constant region of tefibazumab may not have been optimal in the human host. In another approach, Tkaczyk and colleagues isolated human MAb 11H10 by immunizing VelocImmune mice with the N2-N3 subdomain of ClfA, i.e., the fibrinogen binding domain of ClfA (28, 79). While the binding site of MAb 11H10 on ClfA is not known, this antibody shares similar activities with our anti-N3 antibodies. MAb 11H10 inhibited ClfA interactions with fibrinogen, reduced bacterial agglutination in plasma, and promoted opsonophagocytic killing (28). Yet, passive immunization of BALB/c mice resulted in a modest reduction in bacterial burdens in kidneys as compared to isotype control (~0.5 log reduction 2 days post-blood stream challenge) (28). We observed a greater reduction in bacterial loads with 2A12-mIgG2b (~2.5 log reduction 15 days post-blood stream challenge). While we cannot directly compare mouse and human mAbs, we reported earlier that glycosylation at asparagine 297 in the constant region of hIgG1 is critical for the protection of animals from *S. aureus* blood stream challenges, and antibody glycoengineering can be used to improve the effector functions and augment the therapeutic activity of antibodies (80, 81).

In conclusion, ClfA, a functionally well-characterized antigen of *S. aureus*, represents a great model to decipher antibody activities that correlate with protection against *S. aureus* in infected hosts. Whether non-neutralizing antibodies, such as those with N1-specificity, may exert diluting activity should be taken into consideration. Whether antibodies that bind N2-N3 may be clinically superior to anti-N3 antibodies remains to be determined. But, equally important is the appreciation that successful mAbs must not only neutralize most, if not all, ligand interactions promoted by ClfA, such antibodies must also engage with critical Fc ligands for the effective clearance of bacteria.

## MATERIALS AND METHODS

### Bacterial strains, hybridoma and mammalian cells, and growth media

Wild-type *S. aureus* USA300 LAC, and Newman and its mutants (*clfA, vwb*) were cultured in tryptic soy broth or agar at 37°C. *E. coli* strains DH5α and BL21 harboring recombinant protein expression vector(s) were cultured in Luria-Bertani broth or agar with 100 μg/mL carbenicillin at 37°C. Hybridoma cells were cultured in Iscove’s Modified Eagle’s Medium (IMDM) (Gibco) with 10% fetal bovine serum and 1× Pen Strep (Gibco) antibiotic solution additive. HUVECs were cultured in endothelial cell ready-to-use growth medium (PromoCell) in a humidified incubator at 37°C and 5% CO_2_ injection. All bacterial mutant strains and hybridomas were from our laboratory collection.

### ClfA sequence analyses

ClfA orthologs were identified through a two-step local alignment strategy. First, the full-length ClfA amino acid sequence from *S. aureus* Newman was used as a query in a local BLASTp search against the nr protein database (82). To refine the results, a second BLASTp search was conducted using only the N1-N3 domain of ClfA as the query. Sequences with ≥90% query coverage (*n* = 3,367) were retained for further analysis. These sequences were then subjected to an all-against-all BLASTp comparison, and the resulting similarity matrix was used to construct a sequence similarity network using a custom Python script. One representative sequence from each cluster (*n* = 58) was randomly selected for global alignment using Clustal Omega (83), and the multiple sequence alignment was visualized using ESPript 3.0 (84).

### Production of mAbs against ClfA

mAbs against ClfA-A were generated as described previously (61, 85). Briefly, 8-week-old BALB/c mice were immunized with purified recombinant ClfA-A Newman (100 μg) diluted 1:1 in Freund’s adjuvant by intraperitoneal injection. Animals were boosted on days 21 and 42. Animals were bled on days 31 and 52 and screened for anti-ClfA antibodies. Mice with the highest immune reactivity against ClfA were further boosted with 25 µg ClfA-A in PBS. Mice were harvested after 3 days, and the isolated splenocytes were fused with the mouse myeloma cell line SP2/mIL-6. For selection, hybridomas were screened by ELISA for antigen-positive clones. For antibody production, hybridomas were cultured to a density of 10^6^ cells/mL in IMDM with 10% FBS until 90%–95% confluence.

### Expression and purification of recombinant proteins, IVIG antibodies, and mAbs

Recombinant A domain of ClfA protein (ClfA-A) from genomic DNA of *S. aureus* strains Newman, N315, WU1, and USA300 LAC was amplified using primers ClfA-A-F: CGCGCGGCAGCCATATGAGTGAAAATAGTGTTACGCAATCTGATAGC and ClfA-A-R: GTTAGCAGCCGGATCCCTTTTCGAACTGCGGGTGGCTCCACTCTGGAATTGGTTCAATTTCACC. ClfA-N1, N2, and N3 from Newman were amplified using the primer pairs N1-F: CGCGCGGCAGCCATATGAGTGAAAATAGTGTTACGCAATCTGATAGC, N1-R: GTTAGCAGCCGGATCCTGCCGCTAAACTAAATGCTCTCATTCTAGGC, N2-F: GTTAGCAGCCGGATCCTGCTTTTACATCATCTTTAGTATTTACATAGTCTGTAAATG, N2-R: CGCGCGGCAGCCATATGGTAGCTGCAGATGCACCG, and N3-F: CGCGCGGCAGCCATATGACTTTGACCATGCCCGC and N3-R: GTTAGCAGCCGGATCCCTCTGGAATTGGTTCAATTTCACC, respectively. The resulting gene fragments were cloned into pET15b vector using the *Nde*I *and BamH*I restriction sites in *E. coli* DH5α. To facilitate protein purification, six histidine residues (6-HIS) and Strep-tag II peptide (Strep) consisting of Trp-Ser-His-Pro-Gln-Phe-Glu-Lys were engineered at the N- and C-termini of each gene product (some variants carried both or either one of the tags for purification and ELISA detection). The resulting plasmids were sequenced and transferred to *E. coli* BL21. The resulting strains were propagated in liquid cultures to mid-logarithmic growth before addition of 1 mM isopropyl β-D-1-thiogalactopyranoside to induce the production of recombinant proteins. Cleared cell lysates were passed over Ni-NTA resin followed by Streptactin resin following manufacturer’s instructions. All mAbs were purified from the supernatants of hybridomas cell cultures grown in serum-free Freestyle 293 medium by affinity chromatography over protein G Sepharose (Cytiva) following the manufacturer’s protocol and dialyzed against PBS (pH 7.2) (86). Hybridoma cells producing 2F10-mIgG1 were purified over ClfA-A crosslinked to AminoLink Plus coupling resin (Thermofisher). Similarly, anti-ClfA antibodies present in IVIG (Privigen 10% liquid intravenous immunoglobulin, CSL Behring) were purified over either ClfA-A, N2, or N3 purified variants crosslinked to AminoLink Plus coupling resin. Proteins and antibodies were dialyzed against PBS and kept at 2 mg/mL for subsequent downstream *in vitro* and *in vivo* assays.

### Enzyme-linked immunosorbent assays

ELISA-based assays were used to measure interactions between mAbs and ClfA-A antigen, ClfA-A and ligands as well as to assess the ability of antibodies to block interactions (competition assays). Briefly, to measure binding interactions between two partners, Nunc Maxisorp 96-well plates (Thermofisher) were coated with either 100 ng recombinant antigens (ClfA-A, N1, N2, or N3 variants), 100 ng mAbs, 100 ng human vWF (Sigma), or bacterial cells at 1 × 10^6^ CFU (per well) in 100 mM carbonate/bi-carbonate buffer (pH 9.6) at 4°C overnight (for proteins) and 37°C for 2 hours (for bacterial cells). After blocking the wells with 1% (wt/vol) bovine serum albumin in phosphate buffer containing tween-20 for 1 hour, 100 μL serial dilutions of either test antibodies (20 μg/mL), human fibrinogen (4 mg/mL; Sigma), human complement factor I (20 μg/mL; MiliporeSigma), recombinant ClfA-A proteins (20 μg/mL), or recombinant vWbp (5 μg/mL) were added to the wells. To evaluate C1q binding to mAbs, mouse C1q (20 μg/mL; CompTech, M099) was added to the plates coated with mAbs. To assess the inhibitory activity of antibodies in blocking interactions (competition experiments), 50 μL of serial dilutions of 20 μg/mL test antibodies was mixed with 50 µL of either human fibrinogen (4 mg/mL), human complement factor I (20 µg/mL), vWbp (5 µg/mL) or vWbp plus human vWF, and then added to plates coated with ClfA-A. Bound antibodies or fibrinogen were determined by incubating with HRP-conjugated anti-mouse IgG antibody (Southern Biotech; 1:10,000 dilution) and HRP-conjugated anti-human fibrinogen antibody (Rockland, 1:5,000), respectively. Bound recombinant ClfA-A and N subdomains were detected with HRP-conjugated anti-StrepII antibody (Genscript; 1:5,000 dilution) or anti-His-HRP antibody (Abcam; 1:10,1000 dilution). Bound mouse C1q was detected with anti-mouse C1q antibody JL-1 (Invitrogen) that had been conjugated using the LYNX Rapid Antibody HRP Conjugation Kit (BioRad; 1:50 dilution). Bound complement factor I was detected using rabbit anti-factor I polyclonal antibody (Invitrogen, 1:5,000 dilution) and goat anti-rabbit HRP antibody (Southern Biotech, 1:5,000). For vWF-vWbp-ClfA-A interaction, recombinant ClfA-A carrying only His tag was added to the reaction, and bound vWbp was assessed with anti-StrepII antibody (Genscript; 1:5,000 dilution). To determine if mAbs compete for the same epitope, serial dilutions of unconjugated 1A5, 1C2, and 2A12 were added as a competitor to HRP-conjugated 2A12 (using LYNX Rapid Antibody HRP Conjugation Kit, BioRad) in 5:1 ratio to ELISA plates coated with isolated N3. To evaluate the presence of antibodies against ClfA in human sera, serial dilutions of IVIG were prepared by an initial dilution of 1:10,000 in blocking buffer, followed by seven 1:5 serial dilutions. One hundred microliters of each dilution was added to wells previously coated with 100 ng recombinant ClfA-A or N1, N2, or N3 variants. Binding was detected by incubating with HRP-conjugated anti-human IgG (Promega, 1:10,000) for 1 hour at room temperature. All plates were developed using OptEIA reagent (BD Biosciences), and signals quantified using a plate reader by recording absorbances at 450 nm (*A*_450_). Half maximum titers, defined as the reciprocal of the dilution yielding 50% of the maximal absorbance, were determined using GraphPad Prism 10.

### *In vitro* agglutination assay

The bacterial agglutination assay in plasma was performed as described previously (13, 87). In brief, 10 mL of *S. aureus* Newman grown for 16 hours was centrifuged, followed by wash with PBS and resuspended in PBS (normalized to absorbance at 600 nm [*A*_600_] = 4). Bacterial cells were stained with SYTO 9 (1:500) (Invitrogen) for 15 min away from light. Then the cells were resuspended in 1 mL PBS after two washes, and test antibodies were added at a final concentration of 10 μg/mL. These bacterial suspensions were mixed 1:1 volumetrically with anti-coagulated mouse plasma on a glass slide, followed by 30-min incubation at room temperature. Samples were observed under an inverted fluorescence microscope (Leica), and 10 different fields of view were captured for quantification of agglutinated area using QPath software. To account for variations in size and pattern of agglutinated areas, the data are presented as a box and whisker plot.

### Adhesion assay

The adhesion of *S. aureus* to host cells was quantified by an *in vitro* perfusion assay adapted from reference (88). Briefly, human umbilical vein endothelial cells were expanded in a culture flask and grown to 80% confluency. HUVECs were used within two passages. Furthermore, 50 μL of the cells was transferred into the channels of μSlide VI 0.4 (Ibidi). The cells were allowed to adhere overnight, followed by the addition of Ca^+^ ionophore (Sigma) to induce human von Willebrand factor secretion (2). *S. aureus* Newman cultures were grown to mid-log phase. Cells were washed, resuspended in PBS, and stained with SYTO 9 (1:500), washed again and resuspended in PBS at *A*_600_ = 0.42 to yield ~1 × 10^8^ cells per mL suspension. Test antibodies were added at 10 μg/mL to the bacterial suspension. The resulting mixture of bacteria and antibodies was flowed at 1 mL/min through the microslide channels containing HUVECs for 15 min using a peristaltic pump. The microslide channels were subsequently washed with PBS at 1 mL/min for 15 min and allowed to dry. Bound staphylococcal cells were visualized under an inverted fluorescence microscope (Leica), and 10 different fields of view were captured. Cells were counted in each field of view, and the data presented as percentage of bound cells (PBS treatment set at 100%) using Prism 10 (GraphPad).

### Bacterial survival in mouse whole blood

The replication of bacteria in freshly drawn blood in the presence of test antibodies was measured as described previously (61, 89). Briefly, 10 μg/mL test antibodies were added to 500 μL of freshly drawn BALB/cJ mouse blood anti-coagulated with 10 μg/mL heparin. Furthermore, 50 μL of a bacterial suspension (2.5 × 10^5^ CFU/mL) was added. When noted, the blood was preincubated with 0.01 mM cytochalasin D for 10 min or 15 μg cobra venom factor for 20 min. Otherwise, tubes inoculated with bacteria were incubated at 37°C for 30 min with slow end-to-end mixing. Next, 500 μL SK buffer containing 0.5% saponin, 100 U streptokinase, 50 μg trypsin, 1 μg DNase, and 5 μg RNase in PBS was added to each sample followed by incubation at 37°C for 10 min to release live bacteria from agglutinates, neutrophil extracellular traps, or intracellular compartments. Sample aliquots were plated on agar for CFU enumeration in triplicates, and all experiments were repeated at least twice independently.

### Passive immunization studies

BALB/cJ mice (6–8 weeks old; 50% female, 50% male) were immunized with test antibodies (5 mg/kg) via intraperitoneal injection. Mice were anesthetized with isoflurane, and sublethal 5 × 10^6^ CFU of S. aureus were retro-orbitally injected 24-hour post-immunization in groups of 8–12 animals. The health of the mice was monitored for signs of acute disease and by recording weight daily. For CFU enumeration of bacteria in kidneys, animals were killed at day 15 post sublethal challenge. All challenge experiments were repeated at least twice.

### Statistical analyses

Data were analyzed for statistical significance at 95% confidence interval using one-way ANOVA with Tukey’s multiple comparison test between samples and the controls with the exception of the study examining vWF and vWbp interactions with ClfA-A, for which the analysis was performed with the unpaired *t* test (Table 2). Binding assays and competition assays using ELISAs were plotted, and the curve was fitted with non-linear (least squares) regression. Differences in agglutination areas and bacterial replication in whole blood were analyzed using Brown-Forsythe and Welch ANOVA followed by Dunnett’s T3 correction for multiple comparisons. Changes in body weight were reported as the average of the body weights of mice across treatment groups and analyzed using the Kruskal-Wallis test followed by Dunn’s multiple test correction for statistical significance. Bacterial burdens in kidneys of infected mice were compared using one-way ANOVA (mixed-model) with Gaussier-Greenhouse correction followed by Tukey’s multiple comparison test. All data were analyzed with Prism 10 (GraphPad Software, Inc.), and *P* values less than 0.05 were considered significant and marked with asterisks on the graphs.

## SUPPLEMENTAL MATERIAL

Supplemental figures mbio.02197-25-s0001.pdf Fig. S1 to S4.

Supplemental Tables mbio.02197-25-s0002.xlsx Tables S1 and S2.

ASM does not own the copyrights to Supplemental Material that may be linked to, or accessed through, an article. The authors have granted ASM a non-exclusive, world-wide license to publish the Supplemental Material files. Please contact the corresponding author directly for reuse.
