# Supplementary material for: Inhibitory activities of monoclonal antibodies against Staphylococcus aureus clumping factor A
Source: mBio. 2025 Sep 8;16(10):e02197-25. doi: 10.1128/mbio.02197-25 (PMC12505888; doi:10.1128/mbio.02197-25)
Supplement: Supplemental figures — Fig. S1 to S4. [file mbio.02197-25-s0001.pdf]

Supplementary Information For

Inhibitory activities of monoclonal antibodies against *Staphylococcus aureus* Clumping factor A

Biswarup Banerjee, Carla Emolo[1]*, Miaomiao Shi*, Abrar Abdullah Al Fardan, Tonu Pius, Muhammad Shafiul Azam, Molly McAdow[2], Olaf Schneewind†, Dominique Missiakas[#]

Department of Microbiology, Howard Taylor Ricketts Laboratory, The University of Chicago, Lemont, Illinois, USA

Running head: Monoclonal antibodies against *Staph. aureus* ClfA

[#]Address correspondence to Dominique Missiakas, dmissiak@bsd.uchicago.edu

[1,2]Present addresses: [1]GSK, Saint-Amand-les-Eaux, Hauts-de-France, France; [2]Yale School of Medicine, Department of Obstetrics, Gynecology and Reproductive Sciences, New Haven, CT

* Carla Emolo and Miaomiao Shi contributed equally to this work. Author order was determined on the basis of seniority.

Supplementary Figures 1-4

Fig. S1A

```
ClfA-A-NM       SENSVTQSDSASNESKSNDSSSVSAAPKTDDTNVSDTKTSSNTNNGETSVAQNPAQQETT 60
ClfA-A-USA300   SENSVTQSDSASNESKSNDSSSVSAAPKTDDTNVSDTKTSSNTNNGETSVAQNPAQQETT 60
ClfA-A-N315     SENSVTQSDSASNESKSNDSSSVSAAPKTDDTNVSDTKTSSNTNNGETSVAQNPAQQETT 60
ClfA-A-WU1      SENSVTQSDSASNESKSNDSSSINAAPKTDNTNVSDTKTTSNTNNGETSVAQNPAQQETT 60
                **********************:.******:********:********************

ClfA-A-NM       QSSSTNATTEETPVTGEATTTTTNQANTPATTQSSNTNAEELVNQTSNETTFNDTNTVSS 120
ClfA-A-USA300   QSSSTNATTEETPVTGEATTTTTNQANTPATTQSSNTNAEELVNQTSNETTSNDTNTVSS 120
ClfA-A-N315     QSSSTNATTEETPVTGEATTTTTNQANTPATTQSSNTNAEELVNQTSNETTSNDTNTVSS 120
ClfA-A-WU1      QSASTNATTEETPVTGETTTTATNQANTPATTQSSNTNAEELVNQTSNETTSNDTNTVSS 120
                **:**************:***:***************************** ********

ClfA-A-NM       VNSPQNSTNAENVSTTQDTSTEATPSNNESAPQSTDASNKDVVNQAVNTSAPRMRAFSLA 180
ClfA-A-USA300   VNSPQNSTNAENVSTTQDTSTEATPSNNESAPQSTDASNKDVVNQAVNTSAPRMRAFSLA 180
ClfA-A-N315     VNSPQNSTNAENVSTTQDTSTEATPSNNESAPQNTDASNKDVVSQAVNPSTPRMRAFSLA 180
ClfA-A-WU1      VNSPQNSTNAENVSTTQDTSTEATPSNNESAPQSTDASNKDVVNQAVNTSAPRMRAFSLA 180
                *********************************.*********.**** *:*********

ClfA-A-NM       AVAADAPAAGTDITNQLTNVTVGIDSGTTVYPHQAGYVKLNYGFSVPNSAVKGDTFKITV 240
ClfA-A-USA300   AVAADAPAAGTDITNQLTNVTVGIDSGTTVYPHQAGYVKLNYGFSVPNSAVKGDTFKITV 240
ClfA-A-N315     A-AADAPAAGTDITNQLTDVKVTIDSGTTVYPHQAGYVKLNYGFSVPNSAVKGDTFKITV 239
ClfA-A-WU1      AVAADAPAAGTDITNQLTDVKVTIDSGTTVYPHQAGYVKLNYGFSVPNSAVKGDTFKITV 240
                * ****************:*.* *************************************

ClfA-A-NM       PKELNLNGVTSTAKVPPIMAGDQVLANGVIDSDGNVIYTFTDYVNTKDDVKATLTMPAYI 300
ClfA-A-USA300   PKELNLNGVTSTAKVPPIMAGDQVLANGVIDSDGNVIYTFTDYVNTKDDVKATLTMPAYI 300
ClfA-A-N315     PKELNLNGVTSTAKVPPIMAGDQVLANGVIDSDGNVIYTFTDYVDNKENVTANITMPAYI 299
ClfA-A-WU1      PKELNLNGVTSTAKVPPIMAGDQVLANGVIDSDGNVIYTFTDYVDNKENVTANITMPAYI 300
                ********************************************:.*::*.*.:******

ClfA-A-NM       DPENVKKTGNVTLATGIGSTTANKTVLVDYEKYGKFYNLSIKGTIDQIDKTNNTYRQTIY 360
ClfA-A-USA300   DPENVKKTGNVTLATGIGSTTANKTVLVDYEKYGKFYNLSIKGTIDQIDKTNNTYRQTIY 360
ClfA-A-N315     DPENVTKTGNVTLTTGIGTNTASKTVLIDYEKYGQFHNLSIKGTIDQIDKTNNTYRQTIY 359
ClfA-A-WU1      DPENVTKTGNVTLTTGIGTNTASKTVLIDYEKYGQFHNLSIKGTIDQIDKTNNTYRQTIY 360
                *****.*******:****:.**.****:******:*:***********************

ClfA-A-NM       VNPSGDNVIAPVLTGNLKPNTDSNALIDQQNTSIKVYKVDNAADLSESYFVNPENFEDVT 420
ClfA-A-USA300   VNPSGDNVIAPVLTGNLKPNTDSNALIDQQNTSIKVYKVDNAADLSESYFVNPENFEDVT 420
ClfA-A-N315     VNPSGDNVVLPALTGNLIPNTKSNALIDAKNTDIKVYRVDNANDLSESYYVNPSDFEDVT 419
ClfA-A-WU1      VNPSGDNVVLPALTGNLIPNTKSNALIDAKNTDIKVYRVDNANDLSESYYVNPSDFEDVT 420
                ********: *.***** ***.****** :**.****:**** ******:***.:*****

ClfA-A-NM       NSVNITFPNPNQYKVEFNTPDDQITTPYIVVVNGHIDPNSKGDLALRSTLYGYNSNIIWR 480
ClfA-A-USA300   NSVNITFPNPNQYKVEFNTPDDQITTPYIVVVNGHIDPNSKGDLALRSTLYGYNSNIIWR 480
ClfA-A-N315     NQVRISFPNANQYKVEFPTDDDQITTPYIVVVNGHIDPASTGDLALRSTFYGYDSNFIWR 479
ClfA-A-WU1      NQVRISFPNANQYKVEFPTDDDQITTPYIVVVNGHIDPASTGDLALRSTFYGYDSNFIWR 480
                *.*.*:*** ******* * ****************** *.********:***:**:***

ClfA-A-NM       SMSWDNEVAFNNGSGSGDGIDKPVVPEQPDEPGEIEPIPE 520
ClfA-A-USA300   SMSWDNEVAFNNGSGSGDGIDKPVVPEQPDEPGEIEPIPE 520
ClfA-A-N315     SMSWDNEVAFNNGSGSGDGIDKPVVPEQPDEPGEIEPIPE 519
ClfA-A-WU1      SMSWDNEVAFNNGSGSGDGIDKPVVPEQPDEPGEIEPIPE 520
                ****************************************
```

N1
N2
N3

Fig. S1B

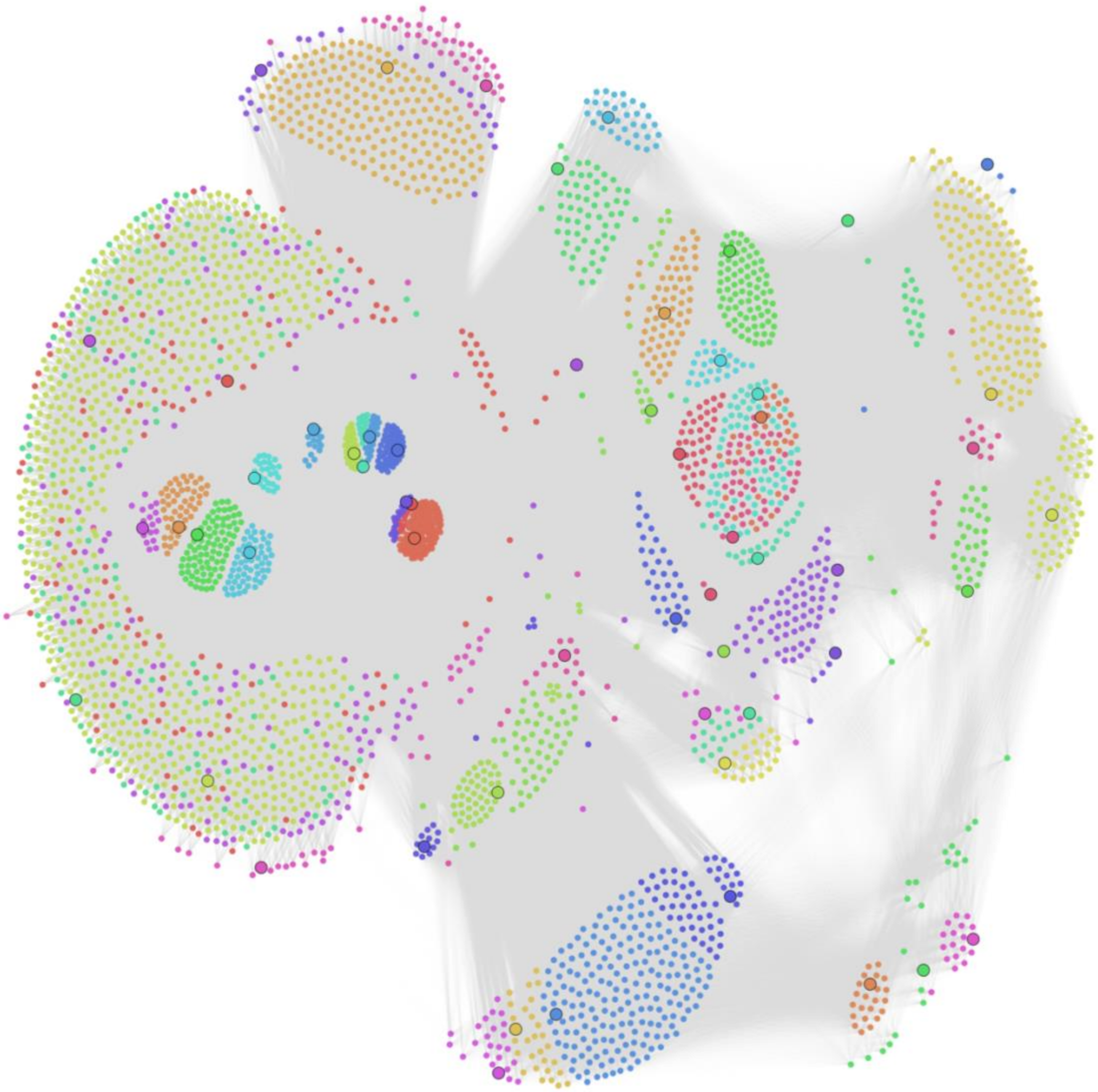

Fig. S1C

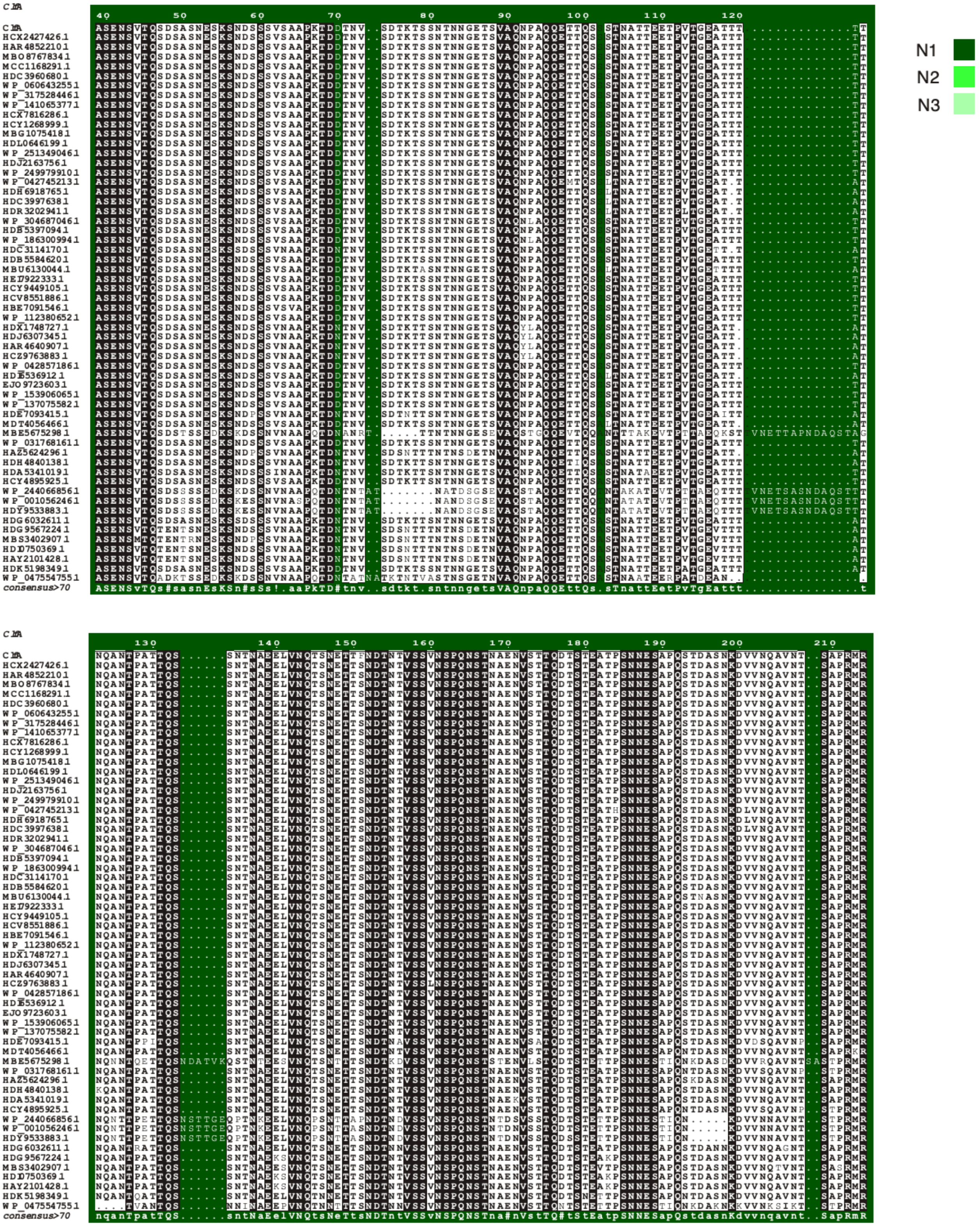

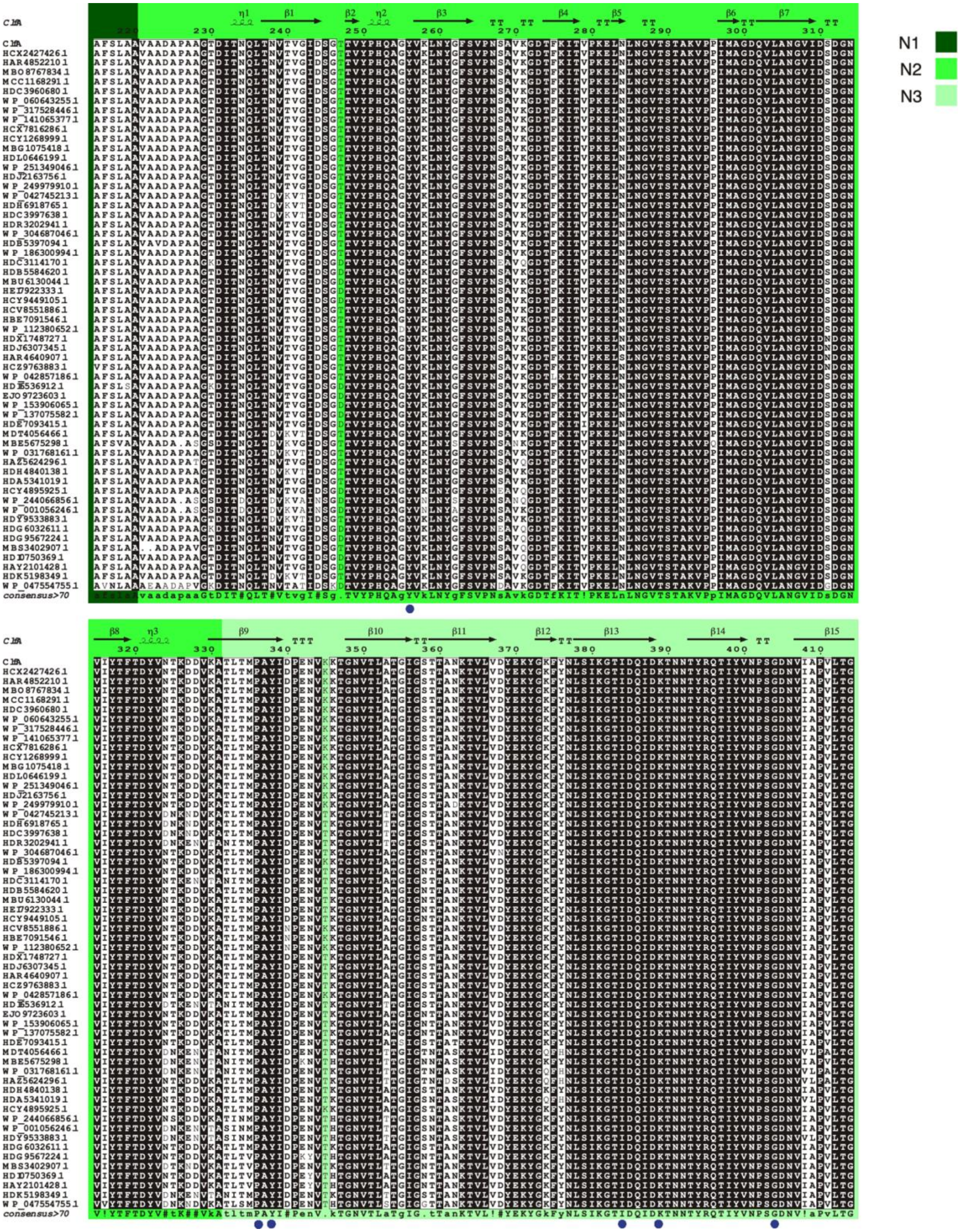
N1
N2
N3

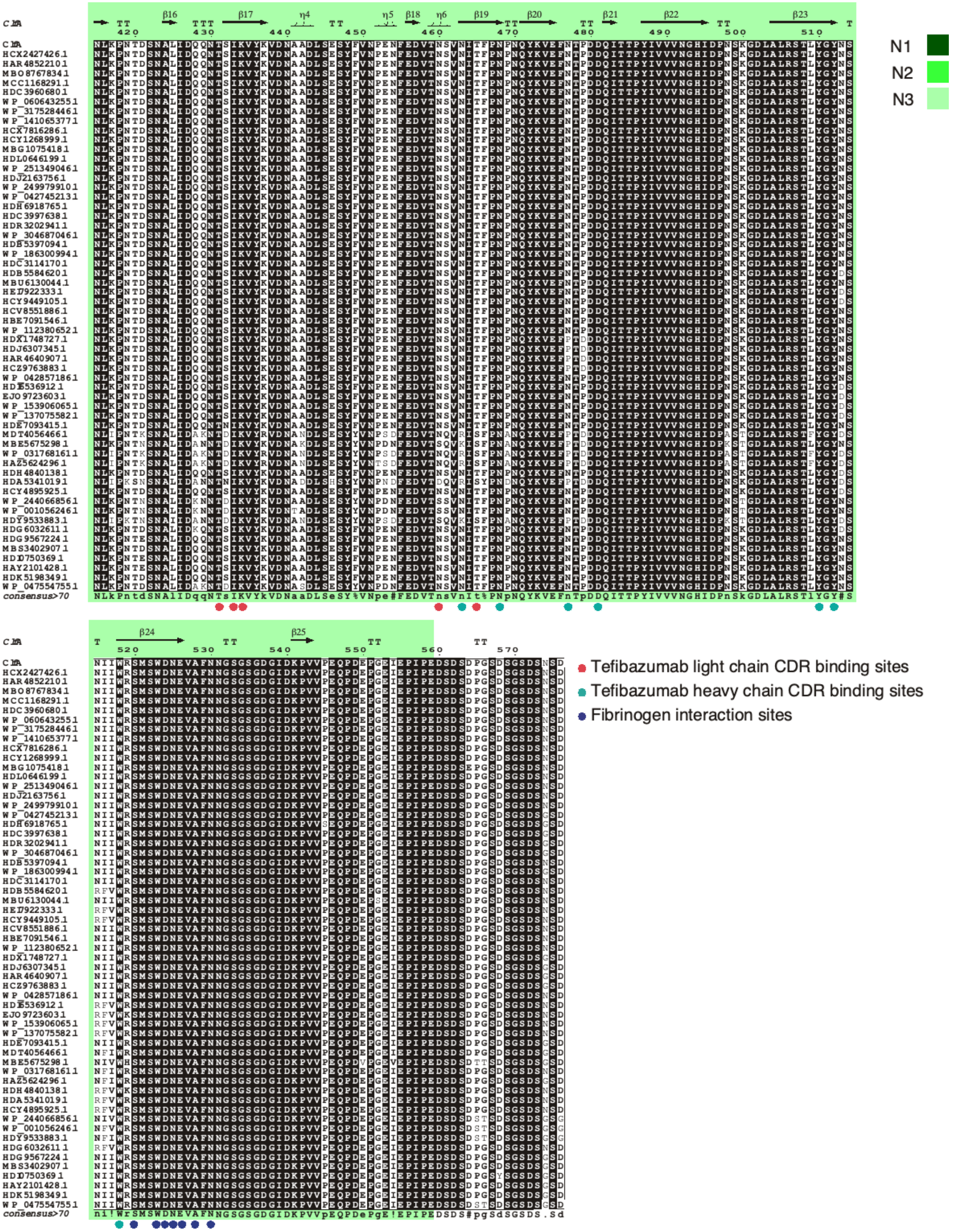
N1
N2
N3
Tefibazumab light chain CDR binding sites
Tefibazumab heavy chain CDR binding sites
Fibrinogen interaction sites

42

Fig. S1. Sequence alignments of ClfA-A proteins. (A) Sequence alignments of ClfA-A domains from strains Newman (NM), USA300 LAC, N315 and WU1 were generated using Clustal Omega (https://www.ebi.ac.uk/jdispatcher/msa/clustalo). The N1, N2 and N3 subdomains are shown in dark, bright and light green, respectively. The conservation score symbols shown below each position in the alignment indicate residues that are: (*),identical; (:), conserved; (.), semi-conserved. The absence of score symbol (blank) indicates non-conserved residues. (**B**) A local alignment search using the *S. aureus* Newman ClfA protein sequence identified ClfA orthologs in the non-redundant database. From these hits, 3367 protein sequences with complete N1–N3 domains were selected. To capture sequence diversity within this region and generate a representative subset for global alignment, a sequence similarity network (SSN) was constructed from all-against-all local alignments of the selected sequences. (**C**) A random representative member of each of the 58 clusters (shown with a dark circle in B) was selected and a multiple sequence alignment was performed to estimate sequence variations in each of N1, N2 and N3 regions of ClfA (dark, bright and light green, respectively). A consensus sequence is indicated at the bottom of aligned segments. Red and teal dots below the residues indicate the binding sites of the light and heavy chains of Tefibazumab, as described in (52). Secondary structures above sequences and amino acids interacting with the C-terminus of the γ chain of fibrinogen (blue dots) are indicated as described in (37).

Fig. S2

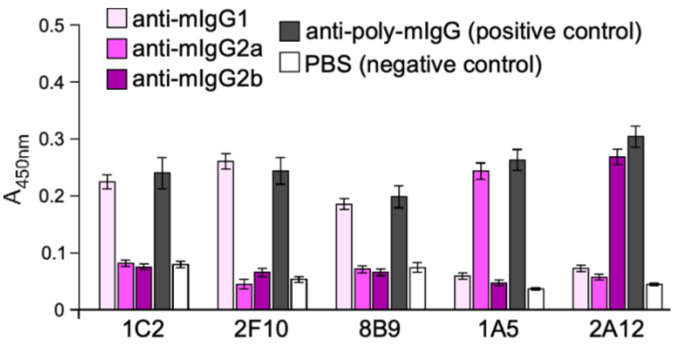

Fig. S2. Isotype determination of experimental mAbs. Candidate antibodies, 1C2, 2F10, 8B9, 1A5 and 2A12 bound to 96-well plates were detected with biotin-tagged anti-mIgG1, 2a, 2b and poly-mIgG, followed by streptavidin-HRP conjugate secondary antibody.

Fig. S3

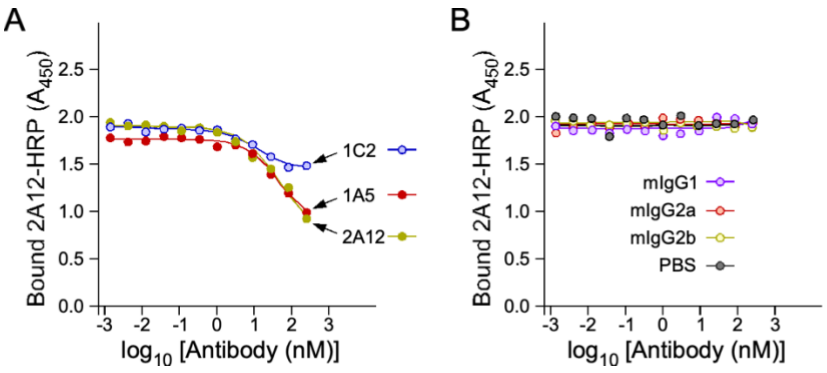

Fig. S3. Competitive ELISA experiment between N3-specific mAbs. HRP labeled 2A12 (2A12-HRP) was mixed with serially diluted unlabeled 1A5, 1C2 and 2A12 (**A**) or isotype control antibodies mIgG1, 2a, 2b or PBS (**B**) and added to ELISA plates coated with the N3 subdomain. Bound 2A12-HRP was assessed by recording absorbance at 450 nm (A_{450}). A representative of three experiments is shown.

Fig. S4

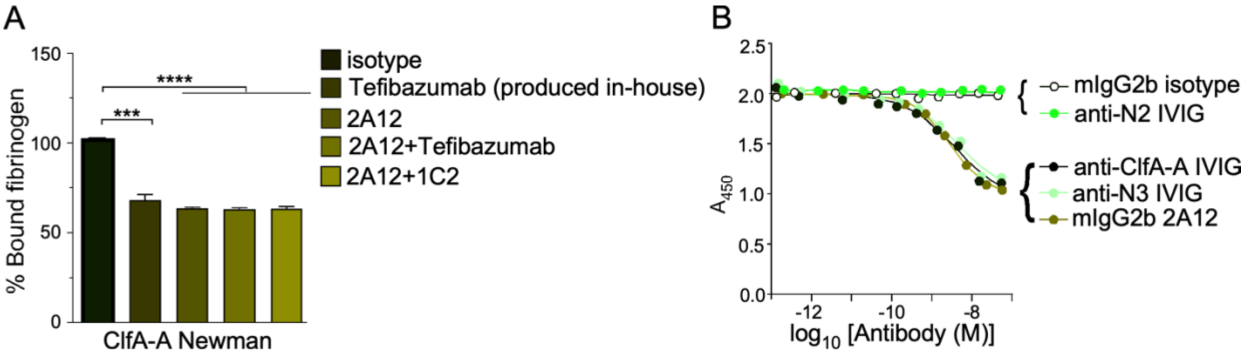

Fig. S4. Competitive ELISA experiment between N3-specific mAbs and fibrinogen. ELISA-plates coated with recombinant ClfA-A from strain Newman were blocked with 1% BSA and 50 µl of serial dilutions of competitor antibody or control were added to the plates along with an equal volume of human fibrinogen (2 mg/ml per well). HRP-conjugated anti-fibrinogen antibody was added to detect the amount of bound fibrinogen followed by TMB coloring substrate and measurement of absorbance at 450 nm. (A) This panel shows the % of fibrinogen remaining bound to ClfA-A in the presence of 25 nM of each antibody alone or in combination (Tefibazumab produced in house, 2A12, 2A12+Tefibazumab, 2A12+1C2). A_{450} values of bound fibrinogen in the presence of isotype control antibodies were set at 100%. Data are represented as mean ± SEM and statistical significance was calculated using One-way ANOVA with Tukey's multiple comparison (****, $P < 0.0001$; ***, $P < 0.001$). (B) Bound fibrinogen over a concentration range of IVIG purified anti-ClfA-A, anti-N2, anti-N3 or 2A12 and isotype control was assessed by recording absorbance at 450 nm (A_{450}). All experiments were performed at least twice.
